# Assessment and attitude of university students about elderly: Preliminary Study

**Published:** 2015

**Authors:** ZA Tabari, FB Ghaedi, JH Hamissi, S Eskandari

**Affiliations:** * Department of Periodontics, Qazvin University of Medical Sciences, Qazvin, Iran; ** Periodontics and Dental Caries Prevention Research Center, Qazvin University of Medical Sciences, Qazvin, Iran; *** Dentist

**Keywords:** attitudes, aging, education, training, geriatric education

## Abstract

**Aims:** The aim of this study was to evaluate the attitude of dental students towards elderly patients. This approach might increase the responsiveness and need of the geriatric dental education within the undergraduate dental students curriculum, which is the persistent necessity for today communities.

**Methods & Materials: **A cross–sectional study was conducted on 201 students who were randomly selected. The investigation was carried out in Qazvin University of Medical Sciences. The attitude of dental students towards elderly was measured with a self-administered questionnaire consisting of an Aging Semantic Differential scale (ASD), which was developed by Rozencranz and Mc Nevin.

**Results: **According to the findings of this study, the students’ attitude remained very positive towards the elderly patients as they showed a strong demand to work with elderly patients. This consisted of a 24 bipolar pair of adjectives that described the attributes of behavioral characteristics thought to be applicable to persons of all ages.

**Conclusion: **According to our finding, the future geriatric dentistry is not towards a weak point in Iran as compared with the undesirable attitudes of dental students in the developed countries.

## Introduction

Increasing the quality of health care is the main great concern in all health care systems [**1**,**2**].And, oral health is a portion of general health and it affects the total happiness of individuals [**3**]. Medical and health literature shows high demands for geriatric care in a world in which health professionals, in particular physicians, tend to exhibit negative attitudes toward the elderly. In some universities, students are dedicated to having trainings and attending the senior people [**4**-**6**].

We are living in an old age population. Ageing has proceeded extremely in developed countries,but in developing countries, it has also begun to present considerable increases [**7**-**8**]. 

Iran has started to come across with the population ageing too. Nevertheless, Iran still has a relatively young population; the amount of elderly being likely to be double in less than 20 years [**9**]. 

According to the report of the United Nations, the statistical projections demonstrated a rapid growth of the elderly population in Iran. While the number of people with 60 years old age and above in Iran were 5.4% in 1975 it will be increasing to 10.5% in 2025 and also 21.7% in 2050 [**10**]. As a matter of fact the total size of Iran population will fail to double in next fifty years, but the number of elderly aged 65 years and over will experience about six-more times increase [**11**].

Rendering to the United Nations, the number of people aged 60 years or older was likely to be 629 million in 2002 and to be develop to nearly a billion by year 2050 [**12**]. The amount of people aged over 60 years will reach up to 21% of the population [**13**,**14**]. The increasing number of elderly people and decreasing rates of edentulous highlight the importance of dental education especially focusing on dental geriatrics [**15**-**17**]. 

Geriatric dental education could be defined as a part of helping pre-doctoral dental curriculum. This deals with special knowledge, attitude and technical skills required in the provision of oral health care to older adults [**18**]. Consciousness of the necessity for dental geriatrics within the undergraduate dental student curriculum has been increased significantly in the western world [**19**,**20**].However, no steps have been taken in this part of the word regarding this matter. Particularly, very little was known about the way the dental students responded to geriatric patients. In order to develop an ability in managing geriatric patients, dental students must undergo educational experiences, development of special clinical skills and a caring attitude towards elderly [**21**].

Undesirable attitudes towards elderly are not unique to dental professionals. The seeming acceptance of edentulous national is as a final result of aging, rather than as a pathological process has resulted in the lack of importance associated with the treatment of dental problems of the aged [**22**]. Numerous studies [**23**-**25**] have shown that the attitudes of health professionals in general are negative towards elderly. 

## Methods and Materials 

**Participants**

This cross–sectional survey was conducted on 201 dental students who were randomly selected in 2008, 2009, 2010, and 2011. All the participants had to be taking courses in either pre-clinic or clinic, if not both

**Instrument and Procedure **

The survey instrument was a structured, hand carried and self-administered questionnaire used for data collection. 

All the students filled out forms of demographic information obtained from them, including personal data as for e.g., gender, age, place of birth and two more questions, one dealing with students’ past experience with a geriatric, either as a provider of care and an elderly family member. The second question asked the students if they wished to work with elderly in future. Their attitudes were measured by using the Aging Semantic Differential scale (ASD) by Rozencranz and Mc Kevin [**26**] (**Table 1**), this being the most widely used instrument in gerontological and geriatric education, to assess the stereotypic attitudes young people have toward older adults. 

**Table 1:** Three factor model of aging semantic differential scale[**19**]

INSTRUMENTAL	INEFFECTIVE
Idle	Busy
Passive	Active
Conservative	Liberal
AUTONOMOUS	DEPENDANT
Disorganized	Organized
Uncertain	Certain
Indecisive	Decisive
PERSONAL ACCEPTABILITY	UNACCEPTABILITY
Uncooperative	Cooperative
Dejected	Helpful
Sad	Happy
Unpleasant	Pleasant

This scale consisted of 24 bipolar pairs of adjectives that described attributes of behavioral characteristics thought to be valid to all persons with different ages. For our study, we only used ten bipolar pairs (**Table 2**). The ASD measured attitudes were on three scales. The three attitudinal dimensions were designated parenthetically by the representations below: (A) Instrumental-Ineffective (I-I) It represented the capability of actively pursuing goals, adaptive to changes. Older people were perceived to be low instrumentality. It consisted of three items Idle-Busy, Passive-Active, and Conservative-Liberal [**24**]. (B) Autonomous-Dependent (A-d) was a measure of self-sufficiency and active participation in social life. It consisted of three bipolar pairs of adjective. Disorganized-organized, uncertain indecisive-decisive [**24**]. (C) Personal Acceptability–Unacceptability (Pa-U) measured the extent to which one was flexible, socially at ease and pleasing to others. It consisted of four pairs of items: Uncooperative-Cooperative, Dejected-Helpful, Sad-Happy, Unpleasant–Pleasant. Responses to the bipolar pairs were calculated on 5-point Lickert scale [**24**]. We asked students to place check marks along the scales at the points which they considered best in describing the elderly person. Scores ranged from 10-70 and the mid-scale score i.e. 30 was considered neutral.9 Scores less than the mid-scale score were considered representative of the positive attitude while those above mid-scale were measured as negative. 

## Method of analysis 

Data was entered by using the Epi Info computer program after which it was transferred to the SPSS, version 21, and the program for analysis. Univariate analyses were performed with the use of Chi-square test and the variance analysis in 95% confidence level. The attitude scores were evaluated by using means and standard deviation and gender and distribution of students according to the wish to work with elderly, was shown in percentages.

## Results 

**Graph 1:**
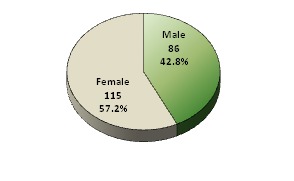
Gender Distribution

Out of 220 dental students, 201 completed the survey, the gender distribution of study population was the following: 115 (57.2%) of these students were females and 86 (42.8%) were males (**Graph 1**). 

The general attitude scores of participants and the mean values were 17.79, which was less than the mid-scale score of 30, for examplethe representative positive attitude of students towards elderly (**Table 2**). This table showed gender distribution, attitude of dental students towards elderly and distribution of students according to whether they wanted to work with elderly people or not (**Table 2**-**Table 4**). 

**Table 2:** Total attitude scores

Statistical values	Total score
Mean	18.2
S.D	5.8
Total no. of scores	1083

The student’s attitude scores with respect to their gender in both male and female showed the same attitude towards elderly as the difference between both genders scores were insignificant (**Table 3**). 

**Table 3:** Attitude scores according to gender

Gender	Statistical values	Total score
Male	Mean	18.2
	S.D	5.8
Female	Mean	17.5
	S.D	4.8
	Level of significance	.351

The distribution of students according to their wish to work with elderly people was 70.6%of the students who wanted to work with elderly and only 16.6% did not want to work with elderly people. All the students had an experience of at least two months in treating elderly patients in the Prosthodontics Department and all students had one old family member at home (**Table 4**). 

**Table 4:** Students distribution according to the desire to work with elderly

Options	Number of students	Percentage
Students who wanted to work	142	70.6
Students who did not want to work	36	16.6
Students who only wanted to work if no other choice was available	23	11.5

## Discussion

The cultural and religious background in Iran is not in favor of leaving elderly people alone, but encourages younger people to take care of their old parents. In developed societies, older people often value their independence and may prefer to live alone [**27**]. In our country, the care of old people in nursing homes or institutions is largely deemed unacceptable by the general public with some exceptions. However, due to recent changes of family size, migration, and also accommodation problems, there is a trend to transfer elders to nursing homes for better care [**28**]. According to our results,it was indicated that students have very positive attitude towards elderly patients. They had a few months of experience of treating the elderly patients in the Department of Prosthodontics [**22**]. 

In this study, a significant difference in attitude of dental students was shown to those with and those without a social contact with the elderly. More positive scores were obtained for students who had at least one old family member at home. In the present investigation, there was no significant difference of attitude between both genders. Possibly, if of a higher sample size it would have shown significant differences between both sexes. The present study was done only on the students of Qazvin University of Medical Sciences. In future research, multicenter studies can be done to reach a consensus, regarding the attitude of dental students towards the elderly in Iran. In the light of that agreement, the curriculum of the undergraduate students could be revised.

## Conclusion

According to our findings, students presented a very positive attitude towards elderly and showed a strong wish to work with the elderly. This study showed that the future of geriatric dentistry was not towards decline, but the only need was to improve the knowledge and skills in the management of the elderly so that the positive attitude could be employed properly to improve the quality of life of elderly. 

**Limitation**

There were some limitations for this study. Future interventions should focus on improving the educational process to provide dental students with positive experiences in dealing with the bio psychosocial concerns of elderly patients. 

**Conflict of interest**

The authors declare that they have no conflicts of interest. 

**Recommendations**

Large nationwide surveys should be carried out to give the most reliable picture of the country. Curriculum of undergraduate students can be revised. 

**Acknowledgment**

We would also like to thank students whose eagerness and willing cooperation without which this survey would have not been possible. There were no funds for this research. We would like to show our appreciation to Mrs. Adeleh Ghodousi for the statistical work. 
